# Predicting Thermodynamic Properties of PBXTHs with New Quantum Topological Indexes

**DOI:** 10.1371/journal.pone.0147126

**Published:** 2016-02-22

**Authors:** Fangzhu Xiao, Guowen Peng, Changming Nie, Limei Yu

**Affiliations:** 1 School of Public Health, University of South China, Hengyang 421001, China; 2 School of Chemistry and Chemical Engineering, University of South China, Hengyang 421001, China; Tsinghua University, CHINA

## Abstract

Novel group quantitative structure-property relationship (QSPR) models on the thermodynamic properties of PBXTHs were presented, by the multiple linear regression (MLR) analysis method. Four thermodynamic properties were studied: the entropy (*S*^θ^), the standard enthalpy of formation (Δ_f_*H*^θ^), the standard Gibbs energy of formation (Δ_f_*G*^θ^), and the relative standard Gibbs energy of formation (Δ_*R*_*G*^θ^). The results by the formula indicate that the calculated and predicted data in this study are in good agreement with those in literature and the deviation is within the experimental errors. To validate the estimation reliability for internal samples and the predictive ability for other samples, leave-one-out (LOO) cross validation (CV) and external validation were performed, and the results show that the models are satisfactory.

## Introduction

Quantitative structure–property relationship (QSPR) remains the focus of many studies aimed at the modeling and prediction of physicochemical properties or biological activities of molecules, because of their convenience and importance for practical use and molecular design when the physicochemical properties or biological activities of compounds are closely related with their structures [1–3].

In QSPR studies, developing topological index is a very crucial step, which is graph theoretical descriptor obtained by transforming molecular structures into the corresponding molecular graphs [4–5]. Since the first topological index *W* was proposed by Wiener in 1947, more and more topological indexes have been constructed because of their simpleness, speediness, and accuracy [6]. Many of them were based on the distance matrixes, such as Balaban index, Hyper-Wiener index, Hyper-Detour index, Detour index, Hosoya index, and Pasareti index. However, these distance matrices consisted of the shortest distances from vertex *i* to all other (*n* -1) vertices in the molecular graphs, and the shortest distance of two adjacent atoms vertex was regarded as ‘‘1”. In fact, the topological space distances is not ‘‘1”, therefore, most of them could not reveal the real connection among atoms, and are not suitable for heteroatom-containing and multiple bond organic compounds [7–9].

Recently, more useful and significant topological indexes have been derived from the molecular structural information and the chemical conditions of atoms, for example, *Lu* index based on the relative electro-negativity and the relative bond length of vertices [10]; the augmented eccentric connectivity index on the ground of the adjacency-cum-distance [11]. At the same time, our group proposed some new topological descriptors, such as *PY*_1_、*PY*_2_ indexes on the basis of space distance matrix, equilibrium electro-negativity and the branching effect [9], *PX*_1_、*PX*_2_ indexes based on topological distance matrix, the branch vertex of atoms and equilibrium electro-negativity [12], *PE* index on the ground of distance matrix and equilibrium electro-negativity[13].

XTH (xanthone) compound, which is the main component of gentianaceaescutellaria stonecrop, is a common folk medicine used as clearing heat, anti phlogosis, liver-protection, cholagogic, detoxification in naxi nationality, tibetan and miao nationality[14]. Due to their wide distribution and important application, xanthonederivants have gained the interest of researchers. For example, PBXTHs (polybrominatedxanthones) are important xanthonederivants[15].

Because the structures of PBXTHs are similar, which have 135 possible structures, according to the number of Br atoms and different replace locations of XTH. If the physical and chemical properties or thermodynamic properties of each PBXTH compounds are determined, it is not realistic in terms of both manpower and material resources. So, QSPRs have been extensively used in molecular structure description and property investigation on PBXTHs and demonstrated obvious advantages.

In this work, as a continuation of our earlier work[8–9, 12–13, 16–18], the new quantum topological indices *XP*_1_、*XP*_2_ of XTH and 135 PBXTHs were constructed combined with the theory of quantum chemistry and topological chemistry. At the same time, the multiple linear regression (MLR) analysis was used to build novel group QSPR models for predicting of the thermodynamic properties (*S*^θ^*S*, Δ_f_*H*^θ^, Δ_f_*G*^θ^Δ and Δ_*R*_*G*^θ^Δ)of XTH and 135 PBXTHs.

## Materials and Methods

### Data Set

All the experimental data of the thermodynamic properties(*S*^θ^, Δ_f_*H*^θ^, Δ_f_*G*^θ^ and Δ_*R*_*G*^θ^*S*) of XTH and 135 PBXTHs used in this work, were obtained from the calculated values in literature [19].

### Construction of new quantum topological indices *XP*_1_、*XP*_2_

The QSPR studies of XTH and 135 PBXTHs were performed in four fundamental stages: (1) Selection of data set; (2) Construction of new quantum topological indices *XP*_1_、*XP*_2_; (3)Multiple linear regression (MLR) statistical analysis; and (4) Model validation techniques. The first as well as the most crucial step is how to exactly extract sufficiently the molecular structure information with numerical format from the molecular graph [20].

**Fig 1:**
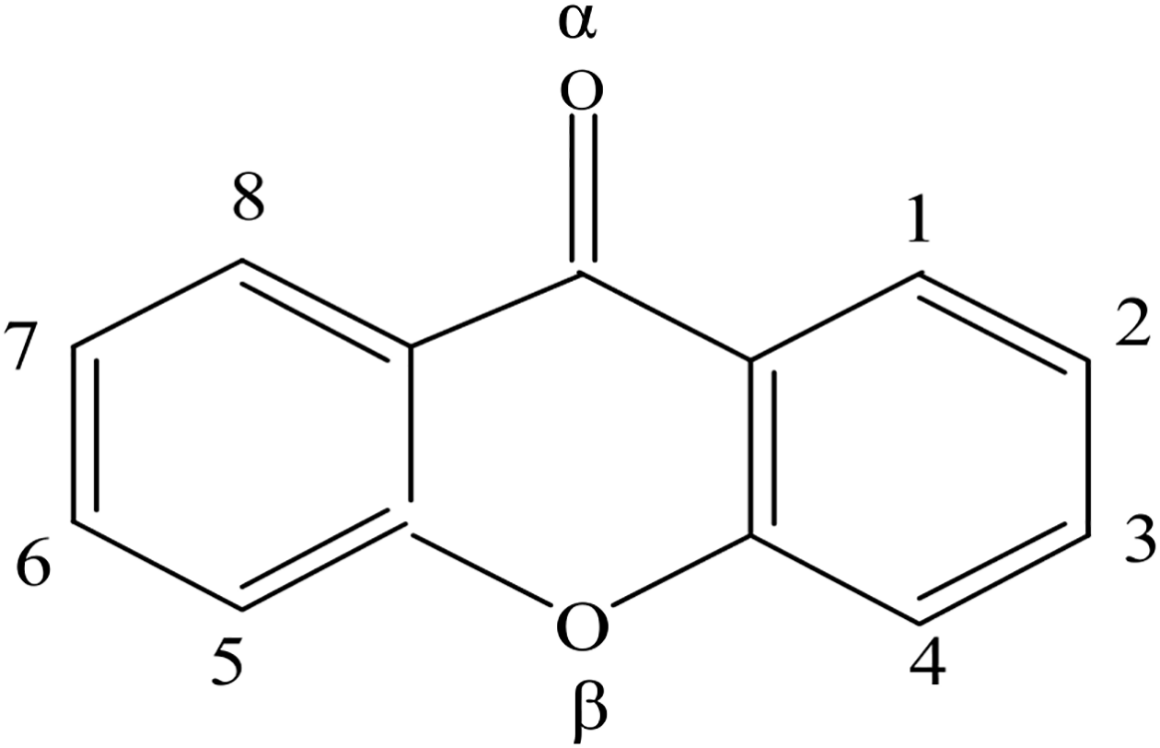
Molecular structure and atomic numbering of XTH.

Structure and atom label of XTH is given in Fig 1:

First, MOPAC 7.0 software was used to optimize the initial geometric parameters of molecular structures of XTH and 135 PBXTHs by constructing and using AM1 semi-empirical quantum chemistry methods. Then, the further geometric configuration optimization and vibration analysis were completed by using Gaussian03 software on the B3LYP/6-31+ G (d) basis set, with the application of density functional theory (DFT). When the stable molecular configuration forming, the potential energy surface scanning method was used to scan all possible bond angle, the dihedral angle, and the corresponding relationship between energy and geometric configuration will be set. On this basis, the spatial topological distance *std*_*ij*_ were calculated between individual atoms of XTH and 135 PBXTHs.

In order to extract the molecular structure information of XTH and 135 PBXTHs sufficiently, we adopt the distance matrix ***D*** and the branching degree matrix ***V*** to descript molecular structure. The distance matrix ***D*** of *n* atoms in a molecule, a square symmetric matrix, can be expressed as *D* = [*d*_*ij*_]_*n*×*n*_. where *d*_*ij*_ is the length of the shortest path between the vertices *i* and *j* in molecular skeleton graph. Instead, in this paper, *d*_*ij*_ was revised by using the spatial topological distance *std*_*ij*_. Therefore, the following distance matrix is 3 ***D*** topological distance matrix ^3^***D***.

D3=[0std12…std1(n−1)std1nstd210…std2(n−1)std2n……………std(n−1)1std(n−1)2…0std(n−1)nstdn1stdn2…stdn(n−1)0]

As one of the main properties of atoms, electro-negativity represents the ability of atoms to obtain or lose electrons when it is in a compound. The larger the electro-negativity of an atom is, the stronger the ability of the atom to attract electrons is. Based on Pauling electro-negativity, the group electro-negativity *x*_G_ can be calculated by the method of stepwise addition[21].

**Fig 2:**
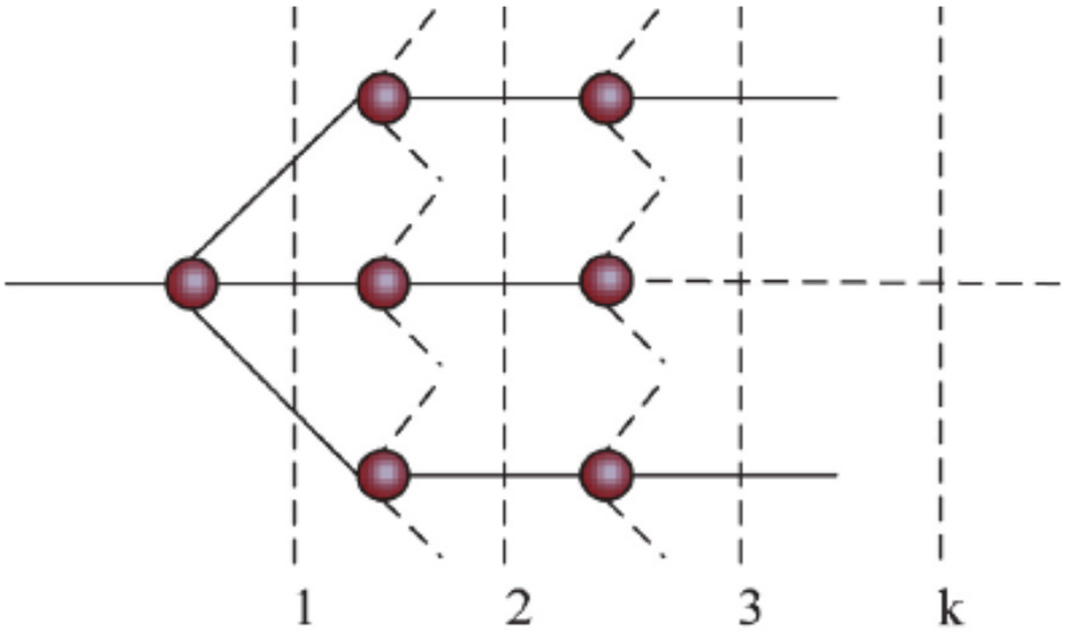
The plot of group structure tree.

The group electro-negativity of a group structural tree is illustrated in Fig 2.

When the group is a single atom, its group electro-negativity is Pauling electro-negativity of this atom. For a group with more than two levels, all the atoms or groups attached to “anchor atom” are weighted equally, which can be expressed as follows [22].

The equilibrium of the first level: $\chi_{0}=\frac{1}{n_{1l}}\sum_{l=1}^{n_{1l}}\chi_{1l}$
The equilibrium of the second level: $\chi_{1l}=\frac{1}{n_{2l}}\sum_{l=1}^{n_{2l}}\chi_{2l}$
……
The equilibrium of the *k-*th level: $\chi_{(k-1)1}=\frac{1}{n_{kl}}\sum_{l=1}^{n_{kl}}\chi_{kl}$

Then, the group electro-negativity χ_*G*_ is defined as:

$$\chi_{G}=\frac{1}{n_{1l}}\sum_{l=1}^{n_{1l}}[\frac{1}{n_{2l}}\sum_{l=1}^{n_{2l}}\ldots (\frac{1}{n_{kl}}\sum_{l=1}^{n_{kl}}\chi_{kl})\ldots ]$$ (1)

For a molecule with an equilibrium structure, the equilibrium electro-negativity of atom *i* is defined as:

$$\chi_{i}=\frac{\chi_{iA}+\sum \chi_{G}}{1+\sum l}$$ (2)

Where χ_*iA*_ is the Pauling electro-negativity for atom *i*, χ_*G*_ is the electro-negativity of group directly attached to atom *i* calculated by Eq (1), and *l* is the group number directly attached to atom *i*.

For a two-level group such as “= CH_2_” and “-CHI_2_”, all of the atom are weighted equally, so that:

$$\chi_{-CH_{2}}=\frac{1}{3}\left(\chi_{C}+2\chi_{H}\right)=\frac{1}{3}\left(2.55\;+\;2\;\times \;2.20\right)\;=\;2.3167$$

And:

$$\chi_{-CHI_{2}}=\frac{1}{4}\left(\chi_{C}+\chi_{H}+2\chi_{I}\right)=\frac{1}{3}\left(2.55\;+\;2.20\;+\;2\;\times \;2.66\right)\;=\;2.5175$$

For a group with more than two levels, all of the atoms or groups attached to the “anchor atom” are weighted equally. For example,

$$\begin{array}{c} \chi_{-CH_{2}CN}=\frac{1}{4}\left(\chi_{C}+2\chi_{H}+\chi_{-CN}\right)=\frac{1}{4}\left(\chi_{C}+2\chi_{H}+\frac{1}{2}\left(\chi_{C}+\chi_{N}\right)\right)\\ =\frac{1}{4}\left(2.55+2\times 2.20+\frac{1}{2}\left(2.55+3.04\right)\right)=2.4363 \end{array}$$

In this paper, the equilibrium electro-negativity matrix ***E*** is established to reflect every atomic chemical environmental change of a molecule, and the matrix ***E*** is defined as following, E = [*χ*_1_ *χ*_2_ … *χ*_n-1_ *χ*_n_]. **T** is the transpose of the matrix (the same below).

In addition, the branching degree matrix ***V*** is established with each atom bonding state and the coupling relationship between atoms, in order to reflect the branching effect of each atom in molecule. The matrix ***V*** is defined as following, *V* = [*v*_1_ *v*_2_ … *v*_n-1_ *v*_*n*_], *v*_i_ is calculated by *v*_*i*_ = *z*_*i*_−*h*_*i*_+1. Where *z*_*i*_ represents the number of valence electron outside the atom nucleus, *h*_*i*_ is the number of hydrogen atoms connecting with atom *i*.

Molecular structure and property are closely related with the atom space effect, the character of the bonding atoms (such as equilibrium electro-negativity) and the branching effect between the atoms. We think that these three factors cooperatively affect the molecular character and property. In this paper, we established a new extension matrix ***S*** on the basis of the topological distance matrix ^3^***D***, the equilibrium electro-negativity matrix ***E*** andthe branching degree matrix ***V***. And the matrix ***S*** is defined: ***M*** = ^3^***D***×***E***×***V***. At the same time, the matrix ***S*** is expressed as following:

S=D3×E×V=[0std12…std1(n−1)std1nstd210…std2(n−1)std2n……………std(n−1)1std(n−1)2…0std(n−1)nstdn1stdn2…stdn(n−1)0]×[χ1χ2…χn−1χn]×[v1v2…vn−1vn]

Then, the correctional matrix ***Q*** is established by Eq (3), on the basis of the extension matrix ***S***.

$$Q=S\times S^{T}$$ (3)

The characteristic values *λ*_Q, n_ of the correctional matrix ***Q*** are calculated using MATLAB, which are arranged from small to big.

In this paper, the new quantum topological indices *XP*_1_、*XP*_2_ will be defined as[9].:

$$XP_{1}=\left|\lambda_{Q}{,}_{\text{min}}\right|\;,\;XP_{2}=\left|\lambda_{Q}{,}_{\text{max}}\right|$$ (4)

Where *λ*_*Q*,min_ is the fisrt characteristic values *λ*_*Q*, 1_ of the correctional matrix ***Q***, and *λ*_Q,max_ is the the *n*-th characteristic values *λ*_*Q*, n_.

**Fig 3:**
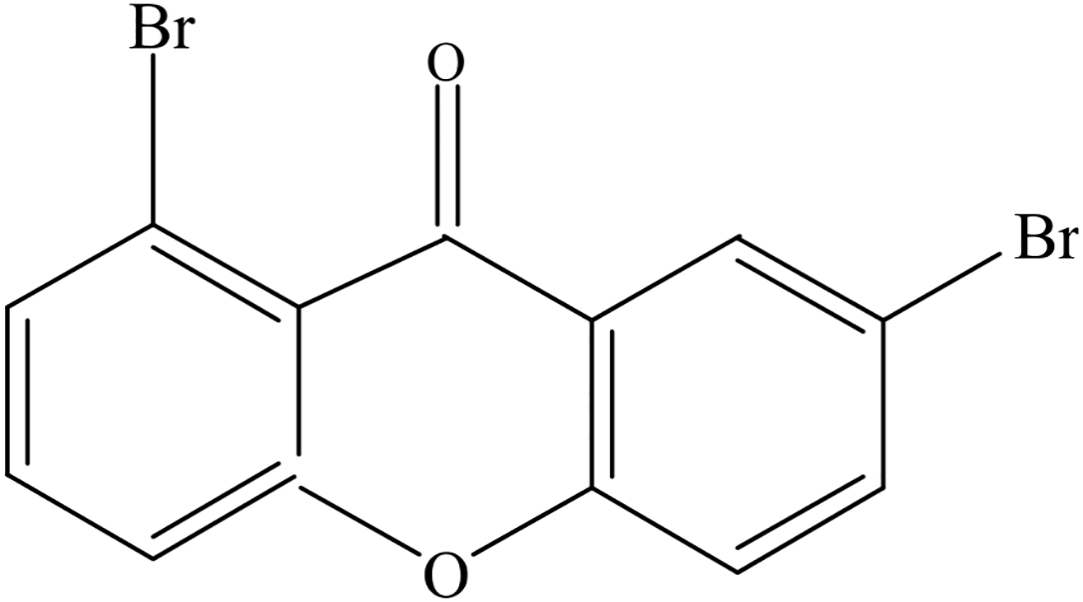
Molecular structure of 2,8-DBXTH.

For example, the molecular structure of 2,8-DBXTH is given in Fig 3. and the correctional matrix ***Q***_2,8-DBXTH_ is given below.

$$Q_{2,8-DBXTH}=\left[\begin{array}{ccccccccccccccccc} 9.6325 & 6.5987 & 8.3568 & 6.3326 & 7.3587 & 6.9985 & 7.0257 & 8.9859 & 9.0257 & 12.3266 & 10.3658 & 11.3582 & 8.3698 & 7.5628 & 8.6329 & 9.3251 & 10.2122\\ 8.3571 & 8.3369 & 11.3201 & 12.3658 & 8.3654 & 10.3222 & 12.0110 & 10.2156 & 9.7555 & 12.3655 & 11.0001 & 10.2254 & 9.1248 & 10.2512 & 9.3562 & 9.2356 & 9.62587\\ 11.2551 & 10.9952 & 9.9852 & 11.0321 & 9.6657 & 9.9523 & 11.0275 & 11.3214 & 10.2255 & 11.0215 & 10.0189 & 9.8952 & 10.2525 & 9.5986 & 10.2578 & 10.2546 & 10.2658\\ 12.0302 & 10.3251 & 10.9958 & 10.2045 & 10.7895 & 11.2546 & 8.3251 & 12.1231 & 11.2126 & 12.2132 & 11.0211 & 9.2012 & 11.0025 & 10.2356 & 11.2456 & 11.3254 & 11.0212\\ 8.3265 & 8.0092 & 9.6697 & 10.0012 & 11.2541 & 10.0018 & 9.3259 & 10.3259 & 10.9956 & 11.2258 & 9.2356 & 8.2659 & 10.2152 & 12.3210 & 10.2365 & 10.2563 & 10.3254\\ 9.2351 & 7.9858 & 10.3258 & 9.8989 & 10.5564 & 10.2129 & 8.9751 & 13.0013 & 12.3568 & 10.3215 & 9.2258 & 9.2012 & 10.2578 & 11.0285 & 12.3546 & 11.3258 & 11.3256\\ 10.0332 & 8.0324 & 11.0322 & 10.2253 & 11.2359 & 11.9513 & 10.2175 & 12.3217 & 13.0201 & 9.8652 & 10.2587 & 11.0215 & 11.3219 & 9.5689 & 11.3258 & 12.5263 & 11.0321\\ 11.0114 & 9.1225 & 10.2254 & 11.2254 & 11.0003 & 11.0215 & 11.2141 & 12.0008 & 11.9586 & 9.1256 & 11.2035 & 10.2578 & 10.2587 & 9.5268 & 10.2569 & 13.2512 & 10.2658\\ 8.3269 & 8.9659 & 11.0002 & 10.3325 & 10.9951 & 9.2568 & 10.2518 & 11.5672 & 11.0245 & 10.2785 & 10.5254 & 11.2515 & 11.0215 & 10.2586 & 10.2987 & 12.0035 & 9.9652\\ 9.0059 & 9.0258 & 10.2121 & 10.0001 & 10.2545 & 9.2564 & 11.2152 & 12.3121 & 10.2578 & 11.3271 & 11.0021 & 10.2129 & 12.0006 & 11.2489 & 11.2546 & 11.9658 & 9.9965\\ 8.3511 & 10.2587 & 11.0368 & 9.2568 & 9.6875 & 10.3256 & 10.2955 & 10.2125 & 11.2151 & 12.5210 & 10.2258 & 12.0321 & 11.0289 & 10.2574 & 10.2548 & 12.2512 & 10.3256\\ 8.9778 & 11.3585 & 12.3325 & 10.2254 & 10.0098 & 9.3695 & 11.2121 & 11.2012 & 10.8985 & 11.3215 & 9.5858 & 11.0021 & 10.0098 & 10.2546 & 11.6548 & 11.6958 & 11.3245\\ 9.0022 & 12.1123 & 11.0332 & 10.5587 & 11.2541 & 13.3121 & 10.6589 & 11.0259 & 11.3258 & 11.0338 & 10.2515 & 9.9562 & 9.5867 & 10.2354 & 11.2154 & 12.3568 & 11.9658\\ 8.9523 & 10.3258 & 8.2257 & 9.9957 & 10.3254 & 10.2356 & 10.5268 & 10.5689 & 11.0208 & 10.2215 & 11.2578 & 12.3251 & 9.1246 & 11.2368 & 12.0221 & 11.9856 & 11.3256\\ 7.2258 & 8.9657 & 9.0056 & 8.5987 & 10.0011 & 12.3211 & 11.3211 & 11.2571 & 10.3118 & 11.0002 & 10.2512 & 11.8595 & 9.1978 & 12.3258 & 13.0005 & 12.0036 & 10.2586\\ 8.2369 & 8.5662 & 10.3278 & 9.3359 & 10.2125 & 10.2113 & 10.2312 & 12.3215 & 9.5687 & 10.0017 & 11.2589 & 11.0017 & 10.2587 & 10.2587 & 12.6589 & 11.6985 & 11.2351\\ 9.3368 & 7.2655 & 8.3365 & 9.0012 & 9.5875 & 10.2103 & 8.2951 & 12.3895 & 9.5235 & 8.9526 & 12.2123 & 9.8945 & 10.2132 & 9.4569 & 11.2548 & 10.2512 & 12.0210 \end{array}\right]$$

The characteristic values *λ*_*Q*_ of the correctional matrix ***Q***_2,8-DBXTH_ are -9.3541, -6.5978, -4.5987, -2.2512, -1.3575, -1.03279, -0.80915, -0.6308, 5.1485, 7.9062, 11.3299, 16.0541, 19.1825, 21.9817, 22.3654, 36.3718and 58.2157, respectively.

Then, the new quantum topological indices *XP*_1_、*XP*_2_ of 2,8-DBXTH are *XP*_1_ = |-9.3541| = 9.3541, *XP*_2_ = |58.2157| = 58.2157, respectively.

According to the same method, the new quantum topological indices *XP*_1_、*XP*_2_ of XTH and 135 PBXTHswere constructed. The calculation results are shown in Table 1.

**Table 1:** Thermodynamic Data and the Quantum Topological Indices *XP*_1_、*XP*_2_ of XTH and 135 PBXTHs.

No.	Compound	Quantum topological index		*S*^θ^(J.mol^-1^.K)		Δ_f_*G*^θ^(KJ.mol^-1^)		Δ_R_*G*^θ^(KJ.mol^-1^)	
		*XP*_1_	*XP*_2_	Cal. [19]	Pre.	Cal.[19]	Pre.	Cal.[19]	Pre.
1	XTH	5.2125	32.2153	415.52	416.32	22.29	22.17	-	-
2	1	6.9527	46.2547	458.50	458.27	61.72	62.01	22.46	22.42
3	2	7.0002	47.2519	456.95	456.53	39.26	40.05	1.57	1.54
4	3	7.1028	48.0126	456.50	456.72	39.26	39.08	0.00	0.02
5	4	7.3001	48.2354	455.96	455.72	47.09	47.57	7.83	7.80
6	1,2	7.4568	48.3257	498.52	498.99	91.77	91.02	34.82	34.91
7	1,3	7.7321	49.2578	499.25	498.95	81.13	81.67	24.18	24.05
8	1,4	7.8126	49.0239	498.61	499.12	89.17	89.01	32.22	32.51
9	1,5	7.8997	49.5672	498.64	498.39	86.76	86.88	29.81	29.75
10	1,6	7.9221	50.2111	499.78	499.72	79.04	79.11	22.09	22.13
11	1,7	8.0025	50.9516	499.41	499.56	80.98	80.67	24.03	24.01
12	1,8	8.1363	51.0021	505.03	504.65	102.60	102.68	45.65	45.71
13	2,3	8.4967	51.3258	495.42	496.05	67.11	67.20	10.16	10.13
14	2,4	8.5115	52.3261	497.27	497.09	68.00	67.85	11.05	11.11
15	2,5	8.5998	52.8573	497.57	497.61	66.37	66.81	9.42	9.51
16	2,6	8.6958	53.2698	497.75	497.82	58.63	58.61	1.68	1.72
17	2,7	8.9657	56.3215	498.52	498.45	60.16	60.09	3.21	3.25
18	2,8	9.3541	58.2157	494.50	494.95	72.61	72.49	15.66	15.61
19	3,5	9.6789	59.2229	496.74	496.69	65.01	64.98	8.06	8.03
20	3,6	9.9215	59.6783	497.72	497.67	56.95	57.06	0.00	-0.01
21	4,5	10.2516	59.0851	496.03	496.41	73.33	73.38	16.38	16.42
22	1,2,3	13.2315	66.3251	537.72	537.69	121.85	121.79	36.67	36.59
23	1,2,4	13.6872	67.3219	538.85	538.92	120.76	120.81	35.58	35.62
24	1,2,5	13.9925	68.0245	539.02	539.11	117.26	117.31	32.08	32.06
25	1,2,6	14.3216	69.1243	539.75	539.67	109.48	109.41	24.30	24.33
26	1,2,7	14.9638	71.3521	539.39	539.42	111.58	111.62	26.40	26.35
27	1,2,8	15.6213	72.2365	539.77	539.82	134.60	134.67	49.42	50.00
28	1,3,4	16.0216	73.0321	536.80	536.85	116.58	116.62	31.40	31.44
29	1,3,5	16.6752	73.9995	539.16	540.02	106.93	106.85	21.75	21.66
30	1,3,6	17.0215	74.3526	540.91	540.85	99.06	99.65	13.88	13.92
31	1,3,7	17.8526	75.0231	540.48	540.92	100.99	100.93	15.81	15.90
32	1,3,8	18.0002	76.3215	550.38	550.29	121.11	120.99	35.93	35.95
33	1,4,5	18.5625	77.0011	538.57	538.61	115.43	115.05	30.25	30.21
34	1,4,6	18.6157	77.8519	539.65	539.59	107.22	107.26	22.04	22.11
35	1,4,7	19.0256	78.6237	539.70	539.75	109.10	109.13	23.92	23.98
36	1,4,8	19.8979	79.0361	544.83	544.65	130.15	130.39	44.97	44.89
37	1,5,6	20.0016	79.9979	537.73	537.69	112.30	112.25	27.12	27.15
38	1,5,7	20.5237	80.2113	539.85	539.89	107.99	108.01	22.81	22.75
39	1,6,7	20.9992	81.0211	538.79	538.82	107.40	107.33	22.22	22.29
40	2,3,4	21.1257	81.95236	533.19	533.12	103.19	103.22	18.01	17.95
41	2,3,5	21.6152	82.0019	535.71	535.62	93.40	93.37	8.22	8.29
42	2,3,6	21.9971	82.6595	537.65	537.59	85.18	85.22	0.00	0.01
43	2,3,7	22.0615	83.0159	536.85	536.91	87.13	87.21	1.95	1.87
44	2,4,5	22.5887	83.8415	537.60	537.55	94.88	95.01	9.70	9.66
45	2,4,6	22.2516	84.0261	538.48	538.52	86.56	86.62	1.38	1.41
46	2,4,7	22.7215	84.8719	538.34	538.25	88.32	88.29	3.14	3.09
47	2,5,6	23.1112	85.0027	535.88	535.72	92.44	92.37	7.26	7.31
48	3,4,5	23.4251	85.9926	535.15	535.02	99.12	99.08	13.94	13.85
49	3,4,6	23.2516	86.0012	535.91	535.85	90.92	89.97	5.74	5.69
50	1,2,3,4	29.3658	92.3215	576.17	576.19	160.91	161.25	46.63	46.59
51	1,2,3,5	30.2555	92.8897	577.31	577.36	147.98	147.91	33.70	33.72
52	1,2,3,6	30.8889	93.2564	578.59	578.62	140.33	140.25	26.05	25.99
53	1,2,3,7	31.0215	93.8523	578.69	578.61	142.12	142.19	27.84	27.79
54	1,2,3,8	31.2227	93.5698	579.78	579.82	165.01	165.11	50.73	50.65
55	1,2,4,5	31.5698	94.0001	578.20	578.22	147.46	147.38	33.18	33.21
56	1,2,4,6	31.2157	94.5158	579.68	579.63	139.47	139.52	25.19	25.33
57	1,2,4,7	31.8555	94.0559	580.35	580.42	141.01	141.12	26.73	26.66
58	1,2,4,8	32.0568	94.9973	580.68	580.59	163.69	163.55	49.41	49.37
59	1,2,5,6	32.2273	95.0379	577.48	577.61	143.12	143.20	28.84	28.79
60	1,2,5,7	32.5587	95.4531	579.78	579.69	139.14	139.08	24.86	24.81
61	1,2,5,8	32.8789	96.1257	579.73	579.80	162.26	162.31	47.98	47.88
62	1,2,6,7	33.0201	96.0087	578.48	578.52	138.55	138.62	24.27	24.63
63	1,2,6,8	33.5218	96.5212	582.00	582.06	154.60	154.55	40.32	40.27
64	1,2,7,8	33.9533	96.9985	576.56	576.61	166.14	166.11	51.86	51.70
65	1,3,4,5	34.0215	97.5218	576.94	576.88	142.65	142.58	28.37	28.82
66	1,3,4,6	34.5555	97.8529	578.04	578.13	135.15	135.09	20.87	2079
67	1,3,4,7	34.9712	97.9967	577.54	577.65	136.91	136.85	22.63	22.56
68	1,3,4,8	35.0073	98.0251	589.08	589.12	155.90	155.81	41.62	41.85
69	1,3,5,6	35.1258	98.1241	577.96	577.89	133.05	133.11	18.77	18.61
70	1,3,5,7	35.3516	98.3216	580.84	580.79	128.77	128.69	14.49	14.52
71	1,3,5,8	35.4562	98.3265	587.35	587.41	150.41	150.37	36.13	36.11
72	1,3,6,7	35.6598	98.6125	578.68	578.63	128.03	128.11	13.75	13.82
73	1,3,6,8	35.7986	98.7219	590.53	590.47	141.68	141.55	27.40	27.36
74	1,4,5,6	35.8975	98.3261	578.07	578.21	141.26	141.32	26.98	26.87
75	1,4,5,7	35.9526	98.2169	579.88	579.76	137.09	137.12	22.81	22.73
76	1,4,5,8	35.9997	99.0002	584.54	584.56	157.96	157.85	43.68	43.72
77	1,4,6,7	36.0249	99.0516	577.88	577.75	136.23	136.16	21.95	22.00
78	2,3,4,5	36.1257	99.2541	573.54	573.49	130.32	130.41	16.04	16.11
79	2,3,4,6	36.3256	99.3528	574.69	574.71	121.93	121.89	7.65	7.68
80	2,3,4,7	36.4586	99.4597	574.45	574.65	123.72	123.67	9.44	9.51
81	2,3,4,8	36.9523	99.6513	575.31	575.42	143.35	143.42	29.07	29.11
82	2,3,5,6	37.1021	99.3332	574.26	574.31	119.72	119.65	5.44	5.38
83	2,3,5,7	37.3216	100.0021	576.70	576.65	115.48	115.53	1.20	1.11
84	2,3,6,7	37.5411	100.3211	575.02	575.13	32.58	33.16	0.00	-0.01
85	2,4,5,6	37.6997	100.8151	575.32	575.28	121.38	121.42	7.10	7.12
86	2,4,5,7	37.8916	100.2137	578.76	578.79	116.93	116.85	2.65	2.59
87	3,4,5,6	38.2131	100.2541	572.98	573.05	125.32	125.25	11.04	11.13
88	1,2,3,4,5	40.2548	110.3215	615.92	615.86	187.46	187.51	36.24	36.32
89	1,2,3,4,6	40.3254	110.5543	617.45	617.55	179.96	179.88	28.74	28.82
90	1,2,3,4,7	40.6579	110.6325	616.93	616.96	181.82	181.93	30.60	30.66
91	1,2,3,4,8	40.8411	110.7327	614.59	614.62	204.23	204.33	53.01	5305
92	1,2,3,5,6	40.9973	110.8216	615.58	615.63	174.29	174.33	23.07	23.02
93	1,2,3,5,7	41.0002	110.9913	618.83	618.81	170.28	170.19	19.06	19.01
94	1,2,3,5,8	41.3217	111.0216	618.16	618.13	193.50	193.55	42.28	42.33
95	1,2,3,6,7	41.4523	111.2311	616.79	616.82	169.40	169.47	18.18	18.23
96	1,2,3,6,8	41.6235	111.3651	619.91	619.86	185.77	185.83	34.55	34.57
97	1,2,3,7,8	41.6997	111.4587	616.44	616.46	196.89	196.93	45.67	45.72
98	1,2,4,5,6	41.8529	111.6201	617.82	617.75	173.47	173.55	22.25	22.23
99	1,2,4,5,7	41.9937	111.7323	619.81	619.79	170.40	170.33	19.18	19.22
100	1,2,4,5,8	42.0315	111.8237	623.34	623.41	185.99	185.85	34.77	34.78
101	1,2,4,6,7	42.3112	111.9025	618.12	618.17	169.11	169.05	17.89	17.93
102	1,2,4,6,8	42.4598	112.0036	621.68	621.73	184.71	184.75	33.49	33.49
103	1,2,4,7,8	43.0257	112.3210	617.34	617.26	195.62	195.59	44.40	44.37
104	1,2,5,6,7	43.2156	112.4202	615.98	615.95	174.23	174.33	23.01	22.89
105	1,2,5,6,8	43.4312	112.9625	618.58	618.62	189.96	189.94	38.74	38.77
106	1,3,4,5,6	43.5264	113.2123	615.93	615.89	168.90	168.93	17.68	17.73
107	1,3,4,5,7	43.7205	113.4652	618.61	618.72	165.28	165.31	14.06	14.08
108	1,3,4,5,8	43.8619	113.7321	624.46	624.53	185.66	185.68	34.44	34.37
109	1,3,4,6,7	43.9935	113.9201	617.66	617.73	164.13	164.07	12.91	12.88
110	1,3,4,6,8	44.0005	114.0223	627.84	627.91	177.47	177.44	26.25	26.22
111	1,3,5,6,7	44.1257	114.2103	616.28	616.33	164.40	164.43	13.18	13.21
112	1,4,5,6,7	44.3251	114.3002	616.13	616.11	172.76	172.81	21.54	21.50
113	2,3,4,5,6	44.4265	114.4001	612.60	612.62	156.68	156.71	5.46	5.42
114	2,3,4,5,7	44.5003	114.5213	614.66	614.72	152.58	152.62	1.36	1.39
115	2,3,4,6,7	44.6325	114.6007	612.93	612.89	151.22	151.31	0.00	0.01
116	1,2,3,4,5,6	48.3257	120.3216	653.67	653.72	213.60	213.62	25.39	25.41
117	1,2,3,4,5,7	48.9125	120.3215	657.04	657.11	209.70	209.72	21.49	21.45
118	1,2,3,4,5,8	49.0216	120.4659	655.16	655.22	232.21	232.16	44.00	44.05
119	1,2,3,4,6,7	49.3257	120.3697	655.52	655.47	209.13	209.09	20.92	21.05
120	1,2,3,4,6,8	49.5635	120.6231	656.21	656.19	225.15	225.16	36.94	36.88
121	1,2,3,4,7,8	49.9937	120.7239	653.45	653.46	235.63	235.60	47.42	47.38
122	1,2,3,5,6,7	50.3215	120.8957	654.55	654.52	205.89	205.93	17.68	17.72
123	1,2,3,5,6,8	50.4538	121.0031	657.85	657.88	221.25	221.23	33.04	33.08
124	1,2,3,5,7,8	50.6513	121.2325	655.13	655.11	226.81	226.79	38.60	38.63
125	1,2,3,6,7,8	50.7331	122.2368	653.15	653.12	228.34	228.42	40.13	40.11
126	1,2,4,5,6,7	50.8369	123.3212	655.79	655.83	205.37	205.31	17.16	17.09
127	1,2,4,5,6,8	50.9978	123.5565	658.93	658.99	219.37	219.42	31.16	31.21
128	1,2,4,5,7,8	51.0301	123.7213	656.24	656.20	225.15	225.13	36.94	36.89
129	1,3,4,5,6,7	51.3265	123.8895	653.66	653.72	200.35	200.37	12.14	12.06
130	1,3,4,5,6,8	51.4325	123.9979	664.62	664.59	212.46	212.53	24.25	24.23
131	2,3,4,5,6,7	51.6679	124.2125	649.81	649.75	188.21	188.26	0.00	-0.01
132	1,2,3,4,5,6,7	60.3321	130.3257	692.35	692.37	245.59	245.63	0.00	0.00
133	1,2,3,4,5,6,8	60.6645	131.0211	694.78	694.81	260.15	260.21	14.56	14.63
134	1,2,3,4,5,7,8	60.7982	131.3251	693.17	693.21	265.63	265.61	20.04	20.01
135	1,2,3,4,6,7,8	60.9125	131.4258	691.40	691.32	267.16	267.22	21.57	21.48
136	1,2,3,4,5,6,7,8	71.3211	142.3212	727.87	727.89	305.44	305.36	-	-

## Results and Discussion

### Regression analysis

The simplest expression of the fundamentalprinciple of QSPR theory is a linear relationship *P* = *a*+*bX* between a property *P* and the chosen moleculardescriptor *X*, where *a* and *b* are real numbers determinedby a standard least-square procedure[23]. According tothe aforementioned method, the multiple linear regression (MLR) analysis using the new quantum topological indices *XP*_1_、*XP*_2_ was performed for obtaining the QSPR models of the thermodynamic properties (*S*^θ^,Δ_f_*G*^θ^ and Δ_R_*G*^θ^) of XTH and 135 PBXTHs. At the same time, to test the stability of QSPR models, leave-one-out (LOO) cross validation (CV)was carried out. Thefinal QSPR models are conducted as follows:

$$\begin{array}{l} S^{\theta }=\;(235.2121\pm \;10.3254)\;+\;\left(10.2193\;\pm \;1.0027\right)XP_{1}+\;\left(5.2016\pm \;0.6975\right)XP_{2}\\ \;n=136;R=0.9971;R_{CV}=0.9970;S=1.0075;F=1835.2692 \end{array}$$ (5)

$$\begin{array}{l} \Delta_{f}G^{\theta }=\;(168.6956\pm \;8.9631)\;+\;(11.3216\pm \;0.9891)XP_{1}+\;(3.6212\pm \;0.5631)XP_{2}\;\\ \;n=136;R=0.9965;R_{CV}=0.9964;S=1.1987;F=1556.3524 \end{array}$$ (6)

$$\begin{array}{l} \Delta_{R}G^{\theta }=\;(263.3112\pm \;12.5726)\;+\;(9.3205\pm \;0.8123)XP_{1}+\;(6.9854\pm \;0.9146)XP_{2}\\ \;n=134;R=0.9982;R_{CV}=0.9980;S=0.9358;F=2531.2545 \end{array}$$ (7)

Where *n* is the number of data points; *R* is the correlation coefficient; *R*_cv_, S, *F* are the cross-validated correlation coefficient, the standard error of estimate, and the Fisher statistic value, respectively.

Particularly, if the correlation coefficient, the Fisher criterion and the cross-validated correlation coefficient are high, the new quantum topological indices *XP*_1_、*XP*_2_ are better to explain the thermodynamic properties (*S*^θ^,Δ_f_*G*^θ^ and Δ_R_*G*^θ^) of XTH and 135 PBXTHs. From Eq (5) to Eq (7), the high correlation coefficient and the low standard deviation of the model indicate that there are very good correlation between the thermodynamic properties (*S*^θ^,Δ_f_*G*^θ^ and Δ_R_*G*^θ^) of XTH and 135 PBXTHs. The correlation coefficient *R*s(*R*,*R*_adj_ and *R*_CV_) of the three QSPR models are all above 0.99, belongs to the optimal level. And, the high correlation coefficient and cross-validated correlation coefficient demonstrate that the new proposed QSPR modelsare more robust and have increased predictive power. Table 1 gives the predicted (Pre.) values of the thermodynamic properties (*S*^θ^,Δ_f_*G*^θ^ and Δ_R_*G*^θ^) of XTH and 135 PBXTHsusing the Eq (5) to Eq (7).

**Fig 4:**
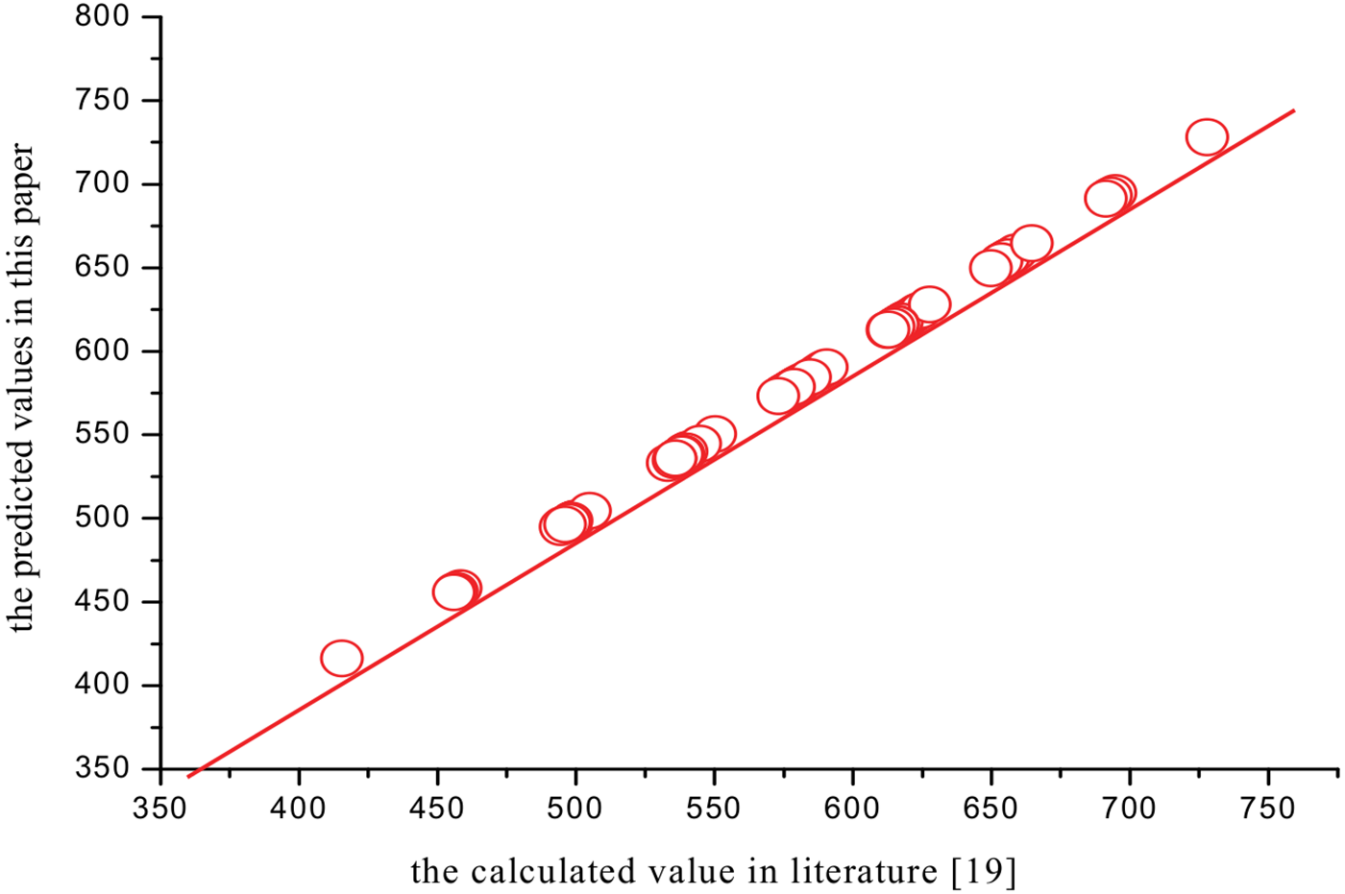
The predicted values versus calculated values in literature[19]of *S*^θ^.

**Fig 5:**
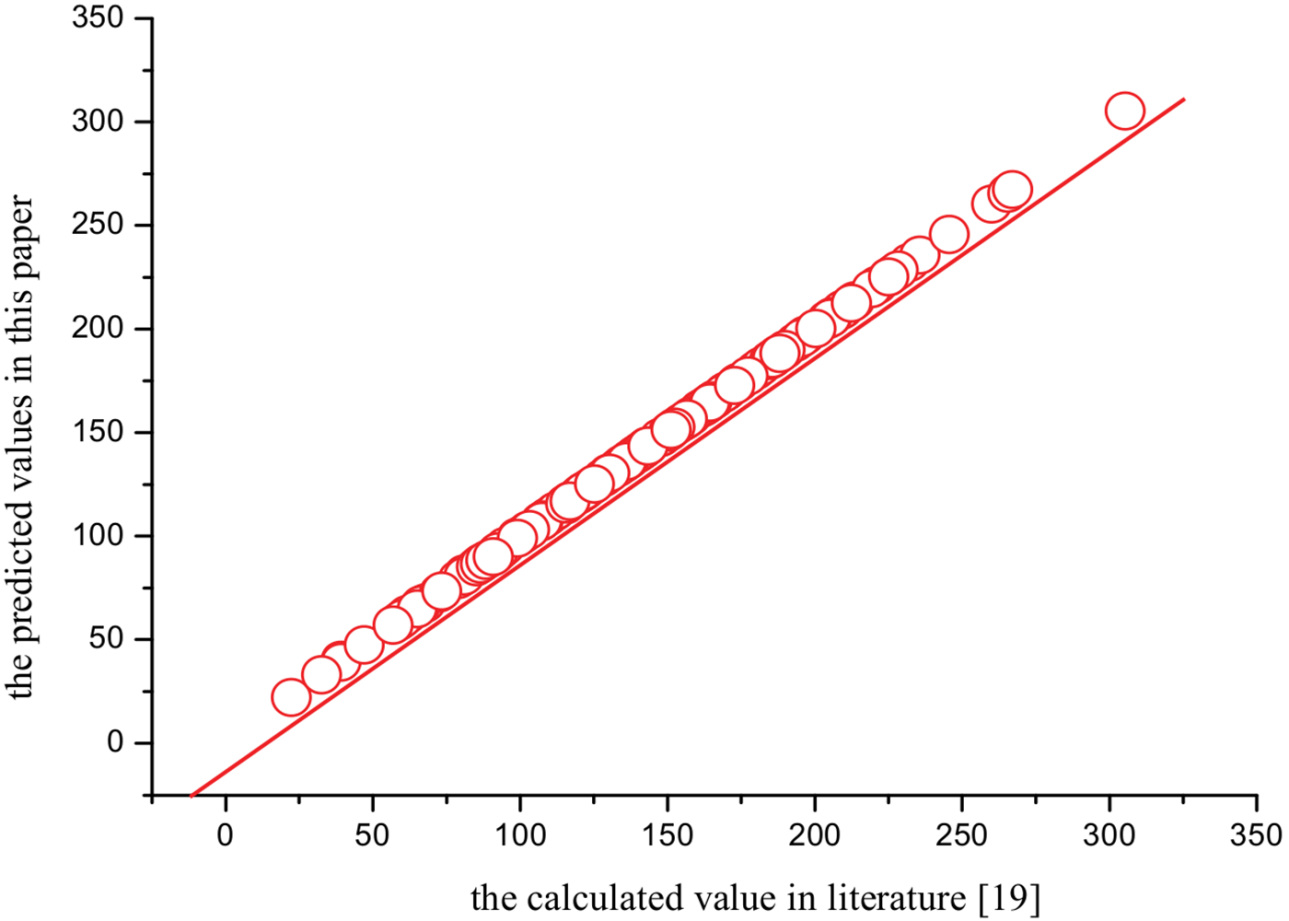
The predicted values versus calculated values in literature[19]of Δ_f_*G*^θ^.

**Fig 6:**
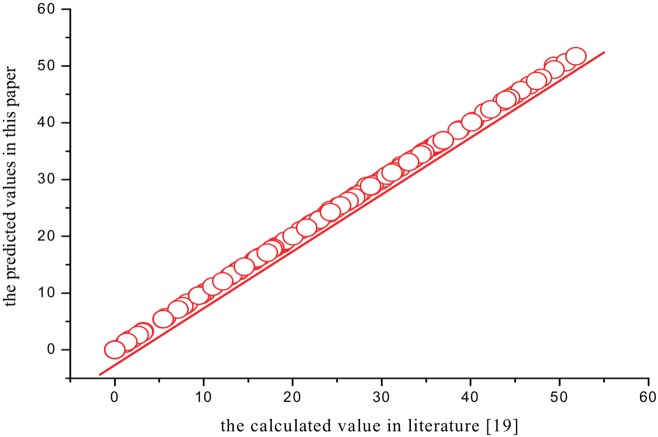
The predicted values versus calculated values in literature[19]of Δ_R_*G*^θ^.

The analysis of plots has shown to be very useful to confirm the quality of a model or to detect the anomalies. The plots of the calculated value in literature[19] versus the predicted values of the thermodynamic properties (*S*^θ^,Δ_f_*G*^θ^ and Δ_R_*G*^θ^) of XTH and 135 PBXTHsare presented in Figs 4–6, which show that they are very close. And the average relative error is only 0.85%, 1.19%, and 0.79%, respectively. All the results show that the three QSPR models have a good predictive power.

### QSPR models cross-validation

All predictive QSPR models require validation to decide whether they can be used to make predictions. If a QSPR model cannot be used to make a prediction, then it is of no practical use. The quality of goodness-of-fit of the models is quantified by the correlation coefficient (including *R*, *R*_adj._, and *R*_CV_), the standard error (*S*), the Fisher statistic value (*F*) and the average relative error (ARE). On the other hand, it is worth mentioning that the models having the best correlation potential need not have the best predictive value[24].

Generally, the most popular validation criterion to explore the robustness of a predictive model is to analyze the influence of each individual object that configures the final equation. This procedure is known as cross-validation (CV) or internal validation by leave-one-out (LOO) [25]. The leave-one-out cross-validations were performed in training test. And each time one compound is left out from the training set, and then the model based on the others is used to predict the compound extracted, that is, a model is built with *n* -1 compounds and the *n-*th compound is predicted. For the test set, the predicted values are obtained from the model using the whole training set. The parameters of the method can play important roles in assessing the performance of QSPR models, which are *S*_*S*_, *D*_*S*_ and *R*_*CV*_[26].

The correlation coefficient for cross-validation (*R*_*CV*_) is then calculated by the following equation:

$$Ss=\sum_{i=1}^{n}\frac{{\left(y_{i,cal}-y_{i,pre}\right)}^{2}}{n-1}$$

$$Ds=\sum_{i=1}^{n}\frac{{\left(y_{i,cal}-y_{i,avg}\right)}^{2}}{n-1}$$

$$R_{CV}=\sqrt{1-\frac{\sum_{i=1}^{n}{\left(y_{i,\text{exp}}-y_{i,pre}\right)}^{2}}{\sum_{i=1}^{n}{\left(y_{i,\text{exp}}-y_{i,avg}\right)}^{2}}}=\sqrt{1-\frac{S_{S}}{D_{D}}}$$

where *n* is the number of compounds included in the QSPR models, *y*_*i*,*cal*_ and *y*_*i*,*pre*_ are the calculated value in literature [19] and the predicted values obtained in this paper using the Eqs (5), (6) and (7), respectively and *y*_*i*,*avg*_ is the average calculated values in literature[19]. From Eq (5) to Eq (7), one can see that the quality of the models for the thermodynamic properties (*S*^θ^, Δ_f_*G*^θ^ and Δ_R_*G*^θ^) of XTH and 135 PBXTHs are satisfactory. And all the values of *R* and *R*_*CV*_ are very close, which shows the good stability and predictivity of the three QSPR models.

In this study, the calculated values in literature [19]of the standard enthalpy of formation Δ_f_*H*^θ^ were studied as test set, and the QSPR model were obtained between the new quantum topological indices *XP*_1_、*XP*_2_ and the calculated values of the standard enthalpy of formation Δ_f_*H*^θ^, according to the topological model Δ_f_*H*^θ^ = a_1_+ a_2_*PX*_1_ + a_3_*PX*_2_. The result shown as following:

$$\Delta_{f}H^{\theta }=\;\left(89.6257\pm \;5.3265\right)\;+\;\left(11.3245\;\pm \;1.3215\right)XP_{1}\;+\;\left(4.5628\pm \;0.\;6325\right)\;XP_{2}$$

$$n\;=136;R=0.9975;R_{\text{adj}}=0.9973;\;R_{\text{CV}}=0.9974;S=1.0938;PRESS=11.2310;\;F=1932.2371$$ (8)

**Fig 7:**
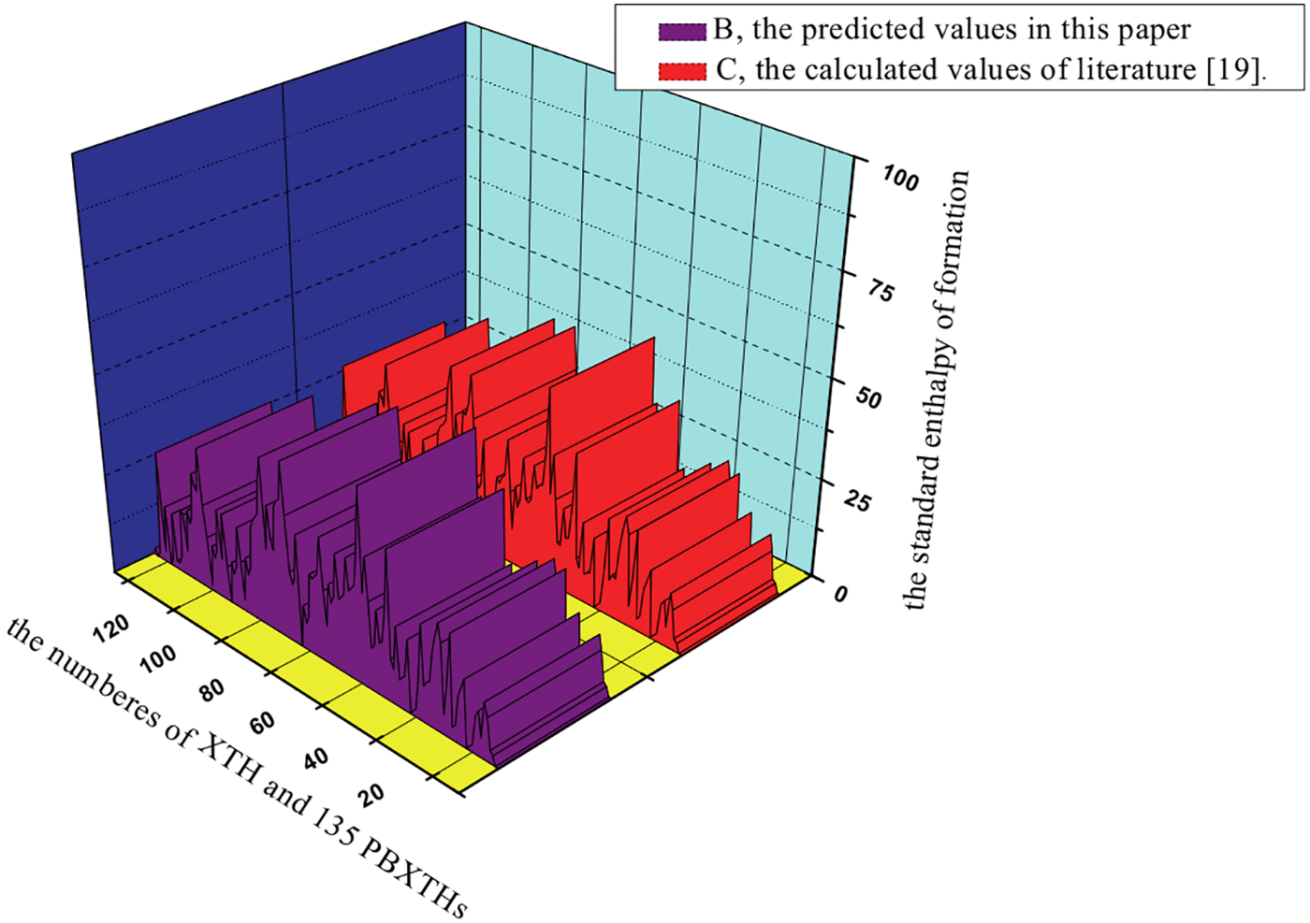
The predicted values versus calculated values in literature[19]of Δ_f_*H*^θ^.

**Fig 8:**
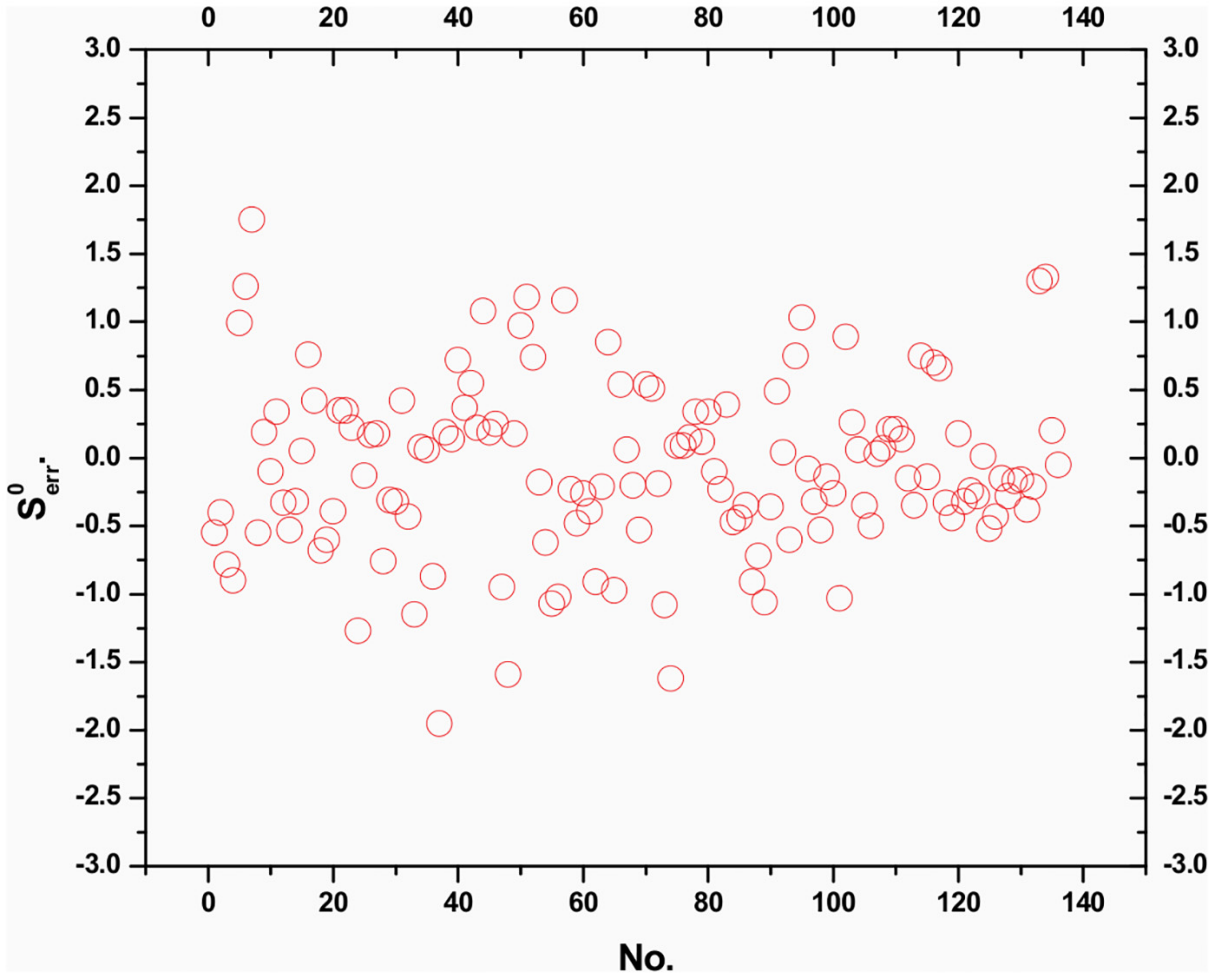
Residuals plot of the predicted values versus calculated values in literature[19]of Δ_f_*H*^θ^.

By comparing the calculated values in literature [10]of the standard enthalpy of formation Δ_f_*H*^θ^,with the predicted values obtained with Eq (8) in this paper, the results show that there are very good correlations. Fig 7 shows that the calculated versus the predicted values obtained with Eq (8) follows a straight line. Fig 7 shows the dispersion as a function of the predicted property. Horizontal lines in this figure indicate the standard deviation limits of ±2S. The residuals exceed seldom the standard deviation of ±2S from Fig 8. Accordingly, from Figs 7 and 8 and the statistical results of Eq (8), it can be concluded that the QSPR model is excellent. And the cross-validated *R*_*CV*_ values (*R*_*CV*_ = 0.9974) are very close to the corresponding *R* value (R = 0.9975). Clearly, the cross-validation demonstrates the final model to be statistically significant.

## Conclusions

1. The new quantum topological indices *XP*_1_、*XP*_2_ efficiently encode the information of chemical environment from the aspect of the equilibrium electro-negativity and the spatial topological distance by revising the traditional distance matrixwith the topological distance matrix ^3^***D***.
2. Based on the new quantum topological indices *XP*_1_、*XP*_2_, quantitative structure −property relationship modelsare built to study the thermodynamic properties(*S*^θ^, Δ_f_*H*^θ^, Δ_f_*G*^θ^and Δ_R_*G*^θ^) of XTH and 135 PBXTHs by the MLR method. Excellent structure−property modelsshow the efficiency of these indices in QSPR studies. In addition, the final model is validated to be statistically reliable and predictive using the general leave-one-out method.
3. Comparison with reference models demonstrate that this new method is very efficient and provides satisfactory results with significant improvements, both in accuracy and stability for predicting the thermodynamic properties of XTH and 135 PBXTHs.
